# Photo Quiz: Carlo Urbani

**DOI:** 10.3201/eid2909.212412

**Published:** 2023-09

**Authors:** Mariano Martini

**Affiliations:** University of Genoa, Genoa, Italy

**Keywords:** SARS, severe acute respiratory syndrome, World Health Organization, Centers for Disease Control and Prevention, history of infectious disease, public health, history of pandemics and epidemics, COVID-19, respiratory infections, severe acute respiratory syndrome coronavirus 2, SARS-CoV-2, coronavirus disease, zoonoses, viruses, coronavirus, Carlo Urbani, Vietnam outbreak, Italy

Who is this person?

Here is a clue: He played a key role in containing the spread of severe acute respiratory syndrome.

A) Ronald Ross

B) David Bruckner

C) Carlo Urbani

D) Li Wenliang

E) Norman Edward Shumway

See next page for the answer.

This is a photograph of Carlo Urbani (1956–2003) (Figure), who was a communicable disease expert for the World Health Organization (WHO) in Hanoi, Vietnam. In 2003, he identified what later became known as SARS and alerted WHO and colleagues elsewhere about his concerns. The disease was originally characterized as pneumonia of unknown origin.

**Figure:**
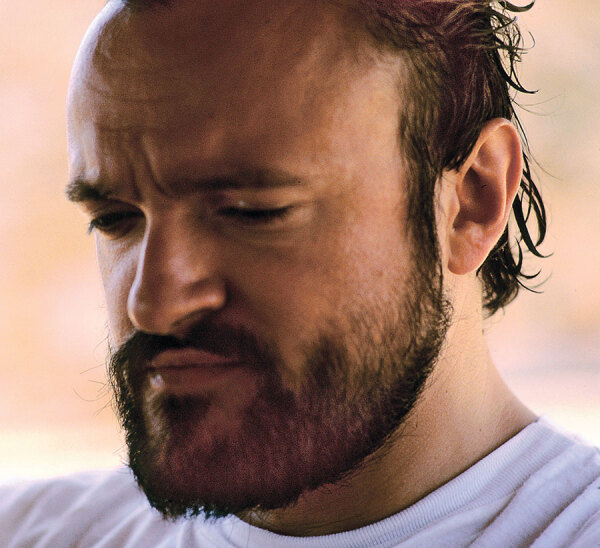
Carlo Urbani

Dr. Urbani was born in Castelplanio, Ancona, Italy, on October 19, 1956. In 1981, he graduated from the University of Ancona with a degree in medicine and surgery and, subsequently, specialized in infectious and tropical diseases at the University of Messina. He worked as a general practitioner and began organizing trips abroad to help poor populations, especially in Africa. In 1993, he became a WHO consultant for control of parasitic diseases and conducted numerous missions in Africa. In 1996, he was appointed as a coordinator of a Médecins Sans Frontières (MSF, of which he was a member) project designed to control parasitic diseases in Cambodia and lived with his entire family in Phnom Penh until 1997. After returning to Italy, he resumed his work as assistant director of the Department of Infectious Diseases at Macerata Hospital, was increasingly involved in MSF missions, and became a WHO consultant for the Western Pacific area. In 1999, he was appointed president of MSF Italia and was a member of the delegation that received the Nobel Peace Prize in Oslo, Norway, that same year.

In 2000, Dr. Urbani made a decision that changed his life; he declined the directorship at Macerata Hospital and accepted an appointment as a WHO expert for the Western Pacific region. He left Italy and moved to Hanoi, Vietnam. Highly aware of the importance of this appointment, which enabled him to assist countries in the region with their efforts to control parasitic diseases, he traveled frequently on missions to critical areas in China, Laos, Cambodia, and the Philippines.

Dr. Urbani was the first WHO doctor to identify SARS in Vietnam, which occurred in a businessman from America who was hospitalized in Hanoi in 2003. SARS cases had been identified earlier in Guangdong Province, China, and exported cases led to hospitalizations in Hong Kong before the first case in Vietnam. One of those patients was hospitalized in Hong Kong on February 17, 2003, after returning from Guangdong Province and had infected close contacts, including healthcare workers and a doctor from that province; the doctor was admitted to an intensive care unit with severe pneumonia on February 22. On February 28, 2003, Dr. Urbani was notified by the Hanoi French Hospital about a patient who was hospitalized with atypical pneumonia. He visited the hospital on Monday, March 3, and realized that he was facing a new, severe, and highly contagious disease. He learned that the patient from the United States had recently stayed at a Hong Kong hotel, where other guests had also been infected; this connection represented the beginning of the international spread of the virus.

Several sources in the scientific literature have indicated that Dr. Urbani understood the situation was critical not only for the hospital staff but also for the entire community because of further contagion risk. However, no one (including Dr. Urbani) would have likely concluded that the situation was critical for the entire community merely from examining the index patient in Vietnam. Indeed, a sporadic case of severe pneumonia in a healthy adult would not raise the alarm of an impending global pandemic, which was what SARS actually became, although it was fortunately contained by rapid and vigorous intervention from WHO. What would have alerted Dr. Urbani to the unusual nature of this disease was the anomalous cluster of severe pneumonia cases that occurred after the index case, especially in young healthcare workers.

Dr. Urbani alerted the government and WHO about the gravity of the situation and possible risks, urging prompt implementation of measures necessary to prevent disease transmission. All infected patients with pneumonia in the Hanoi hospital were isolated, infection control measures were implemented, and the hospital was cordoned off by security guards. On Sunday, March 9, 2003, Dr. Urbani and a WHO representative, Pascale Brudon, called for urgent action against the dangerous new illness, and the Vice Minister of Health in Vietnam immediately assigned a local team to review the situation at the Hanoi French Hospital. A crucial decision was the appointment of 2 experts to help investigate and control the outbreak: Hitoshi Oshitani, WHO’s regional adviser for Communicable Disease Surveillance and Response, arrived on March 10 to head the WHO team, and Tim Uyeki, an influenza expert from the US Centers for Disease Control and Prevention (CDC), arrived on March 11.

On March 11, while flying from Hanoi to Bangkok, Thailand, Dr. Urbani noticed that he had what he believed to be the first symptoms of SARS. Upon his arrival at the Bangkok airport, he warned colleagues who had come to pick him up to keep their distance and asked to be immediately placed in hospital isolation. He asked Scott Dowell from the CDC’s Emerging Infections Program to take 2 swab samples to ensure a good sample was obtained, which became a source of some of the first CDC isolates of SARS-CoV, the cause of SARS.

In Hanoi, Ms. Brudon, Dr. Oshitani, and Dr. Uyeki held an emergency meeting on March 12 with the Vietnam Vice Minister of Health and the director of the National Institute of Hygiene and Epidemiology to discuss recommendations for controlling the outbreak. On March 15, WHO declared that the disease identified by Dr. Urbani was a world health threat, and Ms. Brudon persuaded local authorities to adopt adequate quarantine measures and close the country's ports and borders to curb virus spread. Over the next days, specialists in epidemiology from around the world, including CDC, traveled to Hanoi to join the WHO Vietnam SARS team to help contain and study the outbreak.

On March 29, 2003, after 19 days in isolation, Dr. Urbani died. His dedication to science prompted him to authorize samples of his lung tissues to be collected postmortem and used for research purposes. Dr. Urbani was 1 of ≈80 persons in Vietnam, including many healthcare workers, whose SARS-CoV infections were linked back to the businessman from the United States. At the end of the outbreak, 774 deaths were attributed to SARS worldwide.

The fact that Dr. Urbani immediately reported the first outbreak of SARS in Vietnam was of fundamental importance not only to the local community but also worldwide. His timely action enabled prompt global surveillance of SARS cases, which meant that many patients were identified and isolated before hospital staff could be infected and, above all, before the outbreak of SARS could snowball into a pandemic, which occurred in 2020 for SARS-CoV-2. On April 8, 2003, UN Secretary General Kofi Annan said, “Dr. Carlo Urbani dedicated his life to helping protect and save the lives of others. It was characteristic of his vigilance, professionalism, and expertise that he was instrumental in ensuring an early response by the international community to SARS. Had it not been for his recognition that the outbreak of the virus was something out of the ordinary, many more would have fallen victim to SARS. It was the cruelest of ironies that he lost his own life to SARS while seeking to safeguard others from the disease. Dr. Urbani leaves an inspiring legacy in the United Nations family and the global public health community. For his contribution on the front lines of the fight against disease, he will be remembered as a hero in the best and truest sense of the word.”
